# Patient selection rather than detection alone: Defining the surgical value of radioguided surgery in neuroendocrine tumors

**DOI:** 10.22038/aojnmb.2026.96251.1722

**Published:** 2026

**Authors:** Rıza Deryol

**Affiliations:** Department of Surgical Oncology, Ministry of Health, Istanbul Başakşehir Çam and Sakura City Hospital, Istanbul, Turkey

**Dear Editor**

 We read with great interest the article by Uña-Gorospe et al., “Utility of radioguided surgery in the intraoperative localization of neuroendocrine tumors: Report of 3 cases,” recently published in the *Asia Oceania Journal of Nuclear Medicine and Biology* (1). The authors should be congratulated for presenting a clinically relevant application of radioguided surgery in neuroendocrine tumors (NETs), particularly in cases where intraoperative gamma probe guidance enabled the identification of lesions that were indeterminate, faintly visualized, or not easily detectable by conventional imaging or direct surgical inspection.

 This report highlights the technical feasibility of somatostatin receptor–based radioguided localization. However, we believe that its practical surgical value would be further strengthened by a clearer discussion on patient selection. In daily surgical oncology practice, the key question is not only whether radioguided surgery can detect additional NET lesions, but also in which patients this detection meaningfully changes the operative plan. This distinction is important because NET surgery is highly heterogeneous, ranging from limited local excision to formal oncologic resection with lymphadenectomy or tumor debulking. 

 Therefore, the incremental value of radioguided surgery may be greatest in selected patients with suspected occult disease, equivocal preoperative functional imaging, small subserosal or nodal lesions, recurrent disease, or anatomically challenging locations where visual and tactile feedback is limited (2, 3).

 A related point is the need to link intraoperative radiotracer findings to surgical endpoints. Reporting whether gamma probe findings altered the extent of resection, lymph node dissection, margin assessment, or confirmation of tumor bed clearance would make the technique more interpretable for surgeons. This is particularly relevant because complete macroscopic disease control and margin-oriented resection remain central principles in the management of resectable gastrointestinal NETs (3, 4). Without this operative correlation, radioguided surgery may be perceived primarily as a detection tool rather than a decision-changing adjunct.

 Overall, we congratulate the authors for demonstrating the potential role of radioguided surgery in NET localization. Clarifying the patient selection criteria and the impact of intraoperative decisions would further enhance the practical value of this article for both nuclear medicine physicians and surgical oncologists.
